# Intimate partner violence and current modern contraceptive use among married women in Uganda: a cross-sectional study

**DOI:** 10.11604/pamj.2018.30.85.12722

**Published:** 2018-05-30

**Authors:** Stephen Ojiambo Wandera, Betty Kwagala, Clifford Odimegwu

**Affiliations:** 1 Department of Population Studies, School of Statistics and Planning, College of Business and Management Sciences, Makerere University, Kampala, Uganda; 2 Demography and Population Studies, Schools of Social Sciences and Public Health, University of the Witwatersrand, Johannesburg, South Africa

**Keywords:** Intimate partner violence, modern contraceptive use, sub-Saharan Africa (SSA)

## Abstract

**Introduction:**

This paper examined the relationship between Intimate Partner Violence (IPV) and current modern contraceptive use (MCU) among married women in Uganda.

**Methods:**

We used the 2011 Uganda Demographic and Health Survey (UDHS) data, selecting a weighted sample of 1,307 married women from the domestic violence module. Chi-squared tests and multivariate complementary log-log (clog-log) regressions were used to examine the relationship between IPV and current MCU, controlling for women's socio-demographic factors.

**Results:**

Significant predictors of current MCU (25.3%) among married women were: women's reported ability to ask a partner to use a condom, number of living children and wealth index. The odds of current MCU were higher among women who could ask their partners to use a condom (aOR = 1.87, 95% CI: 1.26-2.78), had more than one child (aOR = 2.05, 95% CI: 1.07,3.93) and were from better wealth indices for example the richest (aOR = 2.52, 95% CI: 1.25-5.08). IPV was not associated with current MCU independently and after adjusting for women's socio-demographic factors.

**Conclusion:**

In Uganda's context, IPV was not associated with current MCU. Interventions to promote MCU should enhance women's capacity to negotiate MCU within union and target women of lower socio-economic status.

## Introduction

Modern contraception is essential for averting maternal deaths through reducing unintended pregnancies and unsafe abortion [1]. Whereas contraceptive prevalence rate (CPR) at the global level was 57% in 2012, sub-Saharan Africa (SSA) lagged behind with a CPR of 24% [2]. In particular, Uganda's modern contraceptive prevalence of 26%, is the lowest in the East African region [3]. Contextual gender relations between men and women influence the latter's reproductive health behavior including fertility regulation or contraception [4]. One of the ways in which men's behaviours influence women's reproductive health outcomes is through intimate partner violence (IPV). Generally, IPV is associated with poor reproductive health outcomes among women [5,6]. Intimate partner violence (IPV) refers to physical, sexual or psychological/emotional violence perpetrated by a husband or intimate partner [7-9]. It includes acts of physical and psychological abuse, forced intercourse and other forms of sexual coercion and controlling behaviors. The prevalence of intimate partner physical, sexual and emotional violence in the last 12 months preceding the 2011 survey in Uganda were 25%, 21% and 33 respectively. About 45% had experienced any of the three forms of violence in the last 12 months from an intimate partner [3]. The association between IPV and modern contraceptive use (MCU) in Africa has mixed findings: some studies report that IPV is associated with increased or decreased contraceptive use, while others report no relationship at all [7]. IPV has been reported to increase MCU in Africa [10, 11] and Nigeria [12]. Elsewhere in sub-Saharan Africa (Cameroon, Kenya, Malawi, Rwanda, Uganda and Zimbabwe using a pooled sample), IPV increased the odds of female controlled modern contraceptive use [10]. Additionally, a systematic review based on longitudinal studies of IPV and contraception also found a significant relationship between IPV and contraceptive use [13]. The explanation for the significant association is that women in such contexts do not wish to have children in conditions that are not conducive [10].

Concerning specific forms of IPV, a study in the Democratic Republic of Congo established that intimate partner sexual violence (IPSV) predicted contraceptive use rather than the combination of intimate partner physical (IPPV) and sexual violence. In the same study, IPSV was a stronger predictor of contraceptive use [7, 10]. In Guinea, women who experience IPV resort to use of injectable, which is not conspicuous to their partners [14]. Other studies have reported that IPV decreases the odds of MCU [15]. IPV was significantly associated with inability to negotiate condom use [16, 17]. In addition, experience of IPV limits control over timing of sexual intercourse, thus limiting effectiveness of some contraceptives such as the condom [7, 18, 19]. A pooled analysis of seven countries in central and west Africa reported that highly tolerating perceptions to domestic violence reduced the odds of using modern contraception [20]. In Egypt, women who experienced physical violence were less likely to use MCU [21]. On the other hand, some studies have found IPV not associated with MCU for example in the Democratic Republic of Congo (DRC) [7], Zambia and Malawi [22]. Since the evidence about IPV and modern contraceptive use is not consistent in Africa, several studies recommend conducting a country specific analyses due to differences in societies and cultures [11]. In Uganda, several studies have assessed predictors of contraceptive use in general [10, 23-26]. One of the studies investigated the association between lifetime modern contraceptive use using the 2003-2006 Uganda Demographic and Health Survey (UDHS) data [10]. A multi-country study using DHS data in 13 African countries (including Uganda) focused more on the interaction between physical IPV and MCU [27]. Therefore, this study aimed at investigating how a combination of the three measures of IPV (physical, sexual and emotional violence) are associated with current MCU.

## Methods

**Data source**: We used the 2011 Uganda Demographic and Health Survey (UDHS) data, with permission from the Demographic and Health Survey (DHS) Program website [28]. Data were collected using a cross-sectional nationally representative survey that employed a stratified two-stage cluster sampling design based on the sampling frame from the 2002 Population and Housing census. Details of the sampling procedure can be accessed in the 2011 UDHS report [3]. A total 2,056 (unweighted cases) ever-married women were selected for the domestic violence module. From this sample, we extracted a weighted sample of 1,307 currently married women. A woman was considered currently married if she was either married or living with a partner [3]. We used the domestic violence weighting variable (d005) found in the UDHS individual women's dataset and the Stata survey (svy) command to apply individual weights during the analyses. Survey weighting was necessary to account for the complex survey design [29].

**Measures of outcome variable**: Modern contraceptive use (MCU) was generated out of variable V313 “current contraceptive use by method type”. It was recoded as a binary outcome (1 = “yes” or 0 = “no”). The first “no method”, the second “folkloric method” and third “traditional method” categories were coded 0 (No) while “modern method” was coded as 1 (Yes). Modern contraceptives included the pill, IUD, injections, diaphragm/foam/jelly and condom, female sterilization, male sterilization and implants. Lactation amenorrhea method cases (5 in number), which were part of the outcome variable were dropped from the analysis [3].

**Measures of explanatory variables**: The main explanatory variable was experience of IPV: physical, sexual or emotional violence. Currently or ever married women were asked a set of questions about violence perpetuated by the current or most recent spouse/partner in the last 12 months preceding the survey. Questions addressing *intimate partner physical violence (IPPV)* were whether the partner ever: a) Pushed, shook, or threw something at her; b) Slapped her; c) Twisted her arm or pulled her hair; d) Punched her with a fist or something that could hurt her; e) Kicked, dragged and beat her up; f) Tried to choke or burn her on purpose; g) Threatened or attacked her with a knife, gun or other weapon. Questions addressing *intimate partner sexual violence (IPSV)*were whether the respondent had ever been: a) Physically forced by the partner to have sexual intercourse with him even when she did not want to; b) Physically forced to perform any sexual acts against her will; c) Forced her with threats or in any other way to perform sexual acts she did not want to *Intimate partner emotional violence (IPEV)* questions were whether the spouse or partner: a) Said or did something to humiliate her in front of others; b) Threatened to hurt or harm her or someone she cared about; c) Insulted her or made her feel bad about herself. An affirmative response to a question was followed with a question about the frequency of the act in the 12 months preceding the survey. A “yes” answer to any of the items in each category constituted physical or sexual or emotional violence [3]. The binary responses (yes or no) from the three categories of questions on physical, sexual and/or emotional violence), were merged into an aggregate measure of IPV [3], coded as 0 = no, and 1 = yes [9]. Women's sexual empowerment was measured by whether a woman could ask the partner to use a condom (0 = no and 1 = yes). Women's socio-demographic factors included were age group (recoded as 15-24, 25-34 and 35+ years), region of residence (Central, Eastern, Northern and Western), place of residence (rural vs urban), religion (Catholic, Protestant, Muslim, Pentecostal/others), current marital status (married or cohabiting), number of living children, education level (no education, primary and secondary or higher), house wealth index (poorest, poorer, middle, richer and richest) and occupation (not working, professional, agriculture and sales).

**Statistical analyses**: We used frequency distributions to describe the characteristics of the women in the sample. This was followed by cross-tabulations with Pearson's chi-squared (χ^2^) tests which examined the associations between modern contraceptive use (MCU), sexual empowerment, IPV and women's socio-demographic factors. The level of statistical significance using p-values was set at p <0.05. Other explanatory variables whose p-values were less than 0.05 or marginally non-significant at bivariate analysis were included in the models. First, complementary log-log (clog log) regression analyses were used to examine the independent association between current modern contraceptive use and the different forms of IPV (physical, sexual and emotional) including IPV. The results are presented in form of un-adjusted odds ratios (un-aOR) in Model 1. Second, women's sexual empowerment measured by ability of a woman to ask her partner to use a condom (Model 2) to the model with IPV as the main explanatory variable. Third, we controlled for women's socio-demographic factors (Model 3). The results for models 2 and 3 were presented in the form of adjusted odds ratios (aOR) and their 95% confidence intervals. All the analyses were weighted to account for complex survey design, clustering and stratification [29]. In order to explore IPV further, we tested for multi-collinearity among variables (results not presented) and found that the mean variance inflation factor (VIF) was 1.52, where household wealth index had the highest (1.98). When the VIF is equal or greater than 5, then multi-collinearity is a problem in the model which means that there was no problem with multi-collinearity among the explanatory variables. However, a correlation test showed a strong positive correlation between age group and the number of living children. We tried to remove age group and kept the number of living children in the model, there was a negligible change in model results (results not presented). Therefore, we put back the variable age group in the model.

## Results

**Descriptive characteristics of the women**: Results in Table 1 show that one in four (25%) of the women considered for the domestic violence model used modern contraceptives. Seven in ten (70%) were below 35 years. Geographical regions were proportionately represented ranging from 19% for Northern to 28% for Central Uganda. The majority (84%) were rural residents and Christians by religion (Catholics 40% and Protestants 29%). Over half (55%) of the women were married. The majority had two or more children (80%), primary or no formal education (78%) and belonged to middle or poor wealth quintiles (59%). Just over half (53%) were engaged in agriculture as their main occupation (Table 1). With respect to sexual empowerment, 75% indicated that they could ask their partner to use a condom. Overall, nearly six in ten (59%) married women experienced any form of IPV (IPPV 41%, IPSV 17% and IPEV 42%) in the last 12 months preceding the survey. Current MCU prevalence was 25% among married women in Uganda. More than half (51%) of the women used injections compared to 18% who used condoms. One in ten women (12%) had undergone female sterilization.

**Table 1:** Socio-demographic characteristics of married women in Uganda

Variables	Categories	Percent (%)	Number (n=1307)
Age group	15-24	29.7	388
	25-34	40.6	531
	35+	29.7	388
Region	Central	28.0	366
	Eastern	26.3	344
	Northern	19.2	251
	Western	26.4	346
Residence	Urban	16.4	214
	Rural	83.6	1093
Religion	Catholic	40.4	528
	Protestant	28.5	373
	Muslim	13.4	176
	Pentecostal	17.7	231
Current marital status	Married	55.3	723
	Cohabiting	44.7	584
Number of living children	0-1	20.1	262
	2-4	44.1	576
	5+	35.9	469
Women's education level	No education	17.0	222
	Primary	60.1	785
	Secondary plus	22.9	299
Household Wealth index	Poorest	18.6	243
	Poorer	19.9	260
	Middle	20.1	262
	Richer	19.5	255
	Richest	21.9	287
Women's occupation	Not working	23.7	310
	Professional	4.0	52
	Agriculture	53.4	698
	Sales	18.9	247
Respondent can ask partner to use condom	No	25.0	327
	Yes	75.0	980
Experienced intimate partner physical violence*	Yes	41.0	537
Experienced intimate partner sexual violence*	Yes	26.5	347
Experienced intimate partner emotional violence*	Yes	41.6	544
Experienced intimate partner violence*	Yes	58.8	769
Current modern contraceptive use	Yes	25.3	331
Current contraceptive method type (N=331)	Pill	8.2	27
	Intra-uterine device (IUD)	2.2	7
	Injections	51.4	167
	Condoms	17.5	57
	Female sterilization	11.8	38
	Implants/ Norplant	8.8	29
	Total		1307

* in last 12 months

**Association between socio-demographic factors, IPV and current modern contraceptive use**: **Table 2** Shows factors that had significant associations with current modern contraceptive use were region (p=0.04), residence (p<0.001), women's level of education (p=0.002) and wealth index (p<0.001). Women's age group and number of living children were marginally significant (p=0.051 and p=0.061 respectively). Modern contraceptive use was highest among married women from Central region (30%), in urban areas (39%), those with secondary or higher education (37%) and the richest wealth quintile (38%). Current MCU was associated with whether the respondent could ask the partner to use a condom (p<0.001). Married women who could ask a partner to use a condom (25%) used MCU more than those who could not (16%). Measures of IPV were not significantly associated with current MCU: IPPV (p=0.41), IPSV (p=0.95), IPEV (p=0.344) and IPV (p=0.74).

**Table 2:** Association between socio-demographic factors and current MCU in among married women in Uganda (DHS 2011)

Variables	Categories	Current MCU (%)	Number	P-value
Age group	15-24	18.8	388	0.051
	25-34	27.0	531	
	35+	29.6	388	
Region	Central	30.2	366	0.035
	Eastern	26.5	344	
	Northern	15.7	251	
	Western	26.0	346	
Residence	Urban	39.4	214	<0.001
	Rural	22.6	1093	
Religion	Catholic	23.3	528	0.093
	Protestant	26.8	373	
	Muslim	33.9	176	
	Pentecostal	20.9	231	
Current marital status	Married	26.7	723	0.381
	Cohabiting	23.7	584	
Number of living children	0-1	14.8	262	0.061
	2-4	27.9	576	
	5+	28.0	469	
Women's education level	No education	15.6	222	0.002
	Primary	23.7	785	
	Secondary or higher	36.8	299	
Household Wealth index	Poorest	10.6	243	<0.001
	Poorer	19.7	260	
	Middle	30.3	262	
	Richer	26.0	255	
	Richest	37.8	287	
Women's occupation	Not working	25.4	310	0.227
	Professional	38.0	52	
	Agriculture	23.2	698	
	Sales	28.7	247	
Respondent can ask partner to use condom	No	15.8	125	<0.001
	Yes	26.3	1182	
Experienced intimate partner physical violence*	No	26.6	770	0.407
	Yes	23.5	537	
Experienced intimate partner sexual violence*	No	25.4	960	0.947
	Yes	25.2	347	
Experienced intimate partner emotional violence*	No	26.7	763	0.344
	Yes	23.4	544	
Experienced intimate partner violence*	No	24.7	538	0.735
	Yes	25.8	769	
	Total	25.3	1307	

* in last 12 months

**Multivariable results**: **Table 3** Presents unadjusted odds ratios (OR) from the complementary log-log regression of current MCU on measures of IPV among married women in Uganda (Model 1). None of the IPV measures (IPPV, IPSV, IPEV and IPV) were associated with current MCU. Since the aim of the study was to investigate the association between IPV and current MCU, the variable IPV, which was an aggregate measure combining IPPV, IPSV and IPEV, was used in the fitting of models 2 and 3 at multivariable levels. In model 2, we controlled for women's ability to ask a partner to use a condom, a measure of sexual empowerment. A woman's reported ability to ask a partner to use a condom was significantly associated with MCU. The odds of current MCU were higher (aOR= 2.07; CI: 1.41-3.06) among women who could ask their partners to use condoms, compared to those that could not. In model 3, we controlled for women's socio-demographic factors. In addition, to sexual empowerment measure (aOR= 1.87; CI: 1.26-2.78), factors associated with current MCU were number of living children and household wealth index. Compared to women who had one child, the odds of current MCU increased with increase in the number of living children (aOR= 2.05, CI: 1.07-3.93 for women with 2-4 children and aOR= 2.30, CI: 1.15-4.59 for women with 5 or more children). The odds of MCU were higher among women in the middle (aOR=2.68; CI: 1.47-4.87), richer (aOR=2.09; CI: 1.08-4.03) and richest (aOR=2.52; CI: 1.25-5.08) wealth quintiles, compared to those from the poorest.

**Table 3:** Adjusted odds ratios (aOR) from regression of current MCU on IPV, controlling for married women’s sexual empowerment and socio-demographic factors in Uganda

	Model (1)+		Model (2)		Model (3)	
Variables	Un-aOR	95% CI	aOR	95% CI	aOR	95% CI
Experienced Intimate Partner Physical Violence (IPPV) in the last 12 months (ref=no)	0.87	[0.62,1.22]				
Experienced Intimate Partner Sexual Violence (IPSV) in the last 12 months (ref=no)	0.99	[0.75,1.32]				
Experienced Intimate Partner Emotional Violence (IPEV) in the last 12 months (ref=no)	0.860	[0.63,1.18]				
Experienced Intimate Partner Violence (IPV) in the last 12 months (ref=no)	1.05	[0.79,1.41]	1.04	[0.78,1.40]	1.12	[0.79,1.58]
Respondent can ask partner to use a condom (ref=no)			2.07***	[1.41,3.06]	1.87**	[1.26,2.78]
**Age (ref=15-24)**						
25-34					1.05	[0.72,1.53]
35+					1.34	[0.84,2.13]
**Region (ref= Central)**						
Eastern					1.17	[0.82,1.65]
Northern					0.89	[0.56,1.43]
Western					1.16	[0.71,1.88]
**Number of living children (ref= 0-1)**						
2-4					2.05*	[1.07,3.93]
5+					2.30*	[1.15,4.59]
**Residence (ref= Rural)**						
Urban					0.71	[0.49,1.05]
**Level of education (ref= No education)**						
Primary					1.32	[0.78,2.23]
Secondary					1.89	[0.87,4.12]
**Wealth quintile (ref= Poorest)**						
Poorer					1.80	[0.98,3.28]
Middle					2.68**	[1.47,4.87]
Richer					2.09*	[1.08,4.03]
Richest					2.52**	[1.25,5.08]
**Population size**	1307		1307		1307	

CI =Confidence Intervals

* p < 0.05-

** p < 0.01-

*** p < 0.001

ref = reference category; Model 1: IPV alone, Model 2: added sexual empowerment; Model 3: added socio-demographic factors; Un-aOR=Un-adjusted Odds Ratios; aOR=adjusted Odds Ratios

## Discussion

This paper aimed at investigating the association between IPV and current modern contraceptive use among married women in Uganda. Only a quarter (25%) of the married women were using modern contraceptives. This was slightly below the national contraceptive prevalence rate of 26% [3]. Nearly six in ten (59%) women reported to have experienced IPV (physical, sexual or emotional) in the last 12 months preceding the survey. Current MCU was associated with women's ability to ask partners to use condoms, number of living children, wealth index but not with IPV. Experiencing IPV in the last 12 months was not associated with current MCU: both at univariable level (Table 2) and multivariable analysis (Table 3). In this study, the results confirmed absence of a direct association between IPV and current MCU among married women in Uganda. As highlighted in the introduction section, findings on the association between IPV and MCU in SSA are mixed [7, 27]. Several studies using DHS data in African countries have reported similar scenarios, with no significant association between IPV and current MCU. IPV was not associated with MCU in the Democratic Republic of Congo [7], Zambia and Malawi [22]. A multi-country study including Uganda also found no association between IPV and MCU, except that its analysis was focused on intimate partner physical violence only [27]. A qualitative study suggested that gender-based violence in general was associated with use of family planning [26]. This paper aimed to include emotional, sexual and physical violence in the measurement of IPV. Forced sex and negotiation for condom use are the critical pathways for the association between IPV and MCU [7]. In this study, intimate partner sexual violence was low (27%) while women's ability to negotiate for condom use was high (75%) among married women in Uganda (Table 1). However, studies which have reported association between IPV and MCU have used life time (ever use of) modern contraceptive use [7, 10], yet our study used current modern contraceptive use. IPV by a current partner is a best assessed with current MCU [7, 10, 13, 16, 30]. Sexual empowerment measured by respondents' ability to ask a partner to use a condom, was significantly associated with MCU. This finding is in agreement with some study in Uganda [23] and in Ghana [31]. The variable is closely related to MCU since the male condom is a contraceptive (as well as a sexually transmitted infections preventive device). Condom use (18%) was the second most popular method used by married women in Uganda, after injections (51%) as indicated in Table 1.

In Uganda's context, women's socio-economic status (measured by household wealth index) seemed to be more important in predicting current MCU, than IPV. From Table 3, the largest effect sizes for the regressions of current MCU were attributed to household wealth index. Women from better wealth indices (middle, richer and richest) were twice more likely to be currently using modern contraceptives. Better wealth status is a protective factor against IPV [32] and a basis for women's sexual empowerment: ability to ask their partners to use condoms. Similar findings have been reported elsewhere, where wealth index increased the likelihood of MCU [23, 33, 34]. In addition, household wealth index, enhances access to modern contraceptives because women are able to afford purchase of modern contraceptive services. As expected, number of living children predicted MCU [33] where women with one or no children had reduced odds of using modern contraceptives. In the Ugandan context, persons in union are expected to have children as soon as possible. This applies to both cohabiting and married persons. In some cases, cohabitation is the initial step towards a formal relationship based on certainty of fertility. Therefore, MCU for women in such situations is limited. Comparison of our findings with other studies should be taken with caution because some studies used different methods [7, 30], datasets, settings, period [10, 30, 33, 35] and pooled data prior to analysis [10]. In some cases, all rather than modern methods of contraception were considered. Some studies focused on IPPV only not all the three measures of IPV [27]. The strength of this paper is its contribution to the discourse of the IPV and current MCU among married women in sub-Saharan Africa. It uses nationally representative sample of women selected for the domestic violence module of the DHS in Uganda. However, the major limitation of the analysis was the cross-sectional nature of the DHS data. It was not possible to establish causal relations between IPV in the last 12 months and current MCU. It was not possible to ascertain whether current MCU preceded IPV or vice versa. Second, the results cannot be generalized to the rest of the sub-Saharan African countries nor the rest of the developed countries. Despite these limitations, the study provides findings that could facilitate targeted response for promotion of MCU by practitioners and could be a basis for designing qualitative studies to explore this subject further.

## Conclusion

Our findings show that women's reproductive rights expressed by a woman's reported ability to ask a partner to use a condom was a significant predictor of current MCU for women in union. Intimate partner violence, women's autonomy with respect to participation in household decision-making and attitudes towards violence, or partners' (controlling) behaviors did not predict MCU. Interventions addressing MCU should therefore promote women's capacity to negotiate MCU with their spouses and target or place emphasis on women from poor households. Since the evidence about IPV and modern contraceptive use is not consistent in Africa, there is need to conduct country specific analyses due to differences in societies and cultures [11].

### What is known about this topic

- The association between physical IPV and lifetime MCU in Uganda is known but not current MCU;
- Findings for the association between IPV and life time or current modern contraceptive use are mixed. Some studies report association-either positive or negative while others found or no associations in African settings.

### What this study adds

- More than half (59%) of married women experienced any of the three forms of IPV and a quarter (25%) of married women in Uganda reported current MCU;
- IPV was not associated with current MCU among married women in Uganda. Instead current MCU was associated with women's negotiation of condom use with their partners, higher parity and better wealth household status;
- Interventions to promote MCU should enhance women's capacity to negotiate MCU within union and target women of lower socio-economic status.
